# Liquid chromatographic determination of enantiomeric purity of [^11^C]methyl-L-methionine and *O*-(2-[^18^F]fluoroethyl)-L-tyrosine by pre-column derivatization with *o*-phthaldialdehyde and *N*-isobutyryl-L-cysteine

**DOI:** 10.1186/s41181-025-00421-z

**Published:** 2026-01-07

**Authors:** Viktória Forgács, Viktória Balla, Viktória Csonka, Dezső Szikra, Dániel Szücs, Enikő Németh, Zita Képes, György Trencsényi, István Jószai

**Affiliations:** Division of Nuclear Medicine and Translational Imaging, Department of Medical Imaging, Faculty of Medicine, University of Debrecen, 98 Nagyerdei St., Debrecen, 4032 Hungary

**Keywords:** [^11^C]methionine, [^18^F]FET, Enantiomeric purity, UPLC, PET

## Abstract

**Background:**

[^11^C]methyl-L-methionine ([^11^C]MET) and *O*-(2-[^18^F]fluoroethyl)-L-tyrosine ([^18^F]FET) are commonly used radiopharmaceuticals in positron emission tomography (PET) for diagnosis of brain tumors. The preparations can be released for human use after the determination of several quality parameters. The enantiomeric purity test is an integral component of the quality control (QC) procedure for these radiopharmaceuticals. In this context, the European Pharmacopoeia monographs recommend thin-layer chromatography (TLC) for the separation of optical isomers of radiolabeled amino acids. To enhance the accuracy and efficiency of the analysis, a liquid chromatographic method should be employed. The aim of this work was to evaluate ultrahigh-performance liquid chromatography (UPLC) for the determination of enantiomeric purity of [^11^C]MET and [^18^F]FET, with the goal of reducing analysis time. Additionally, a pre-column derivatization method was applied using o-phthaldialdehyde (OPA) and N‑isobutyryl‑L‑cysteine (IBLC), which are widely used for the separation of optical isomers of amino acids.

**Results:**

In this work, two novel chromatographic methods were proposed for the determination of enantiomeric purity of [^11^C]MET and [^18^F]FET. The method development involved studying the effects of the type, composition, and acidity of the mobile phase, as well as the flow rate and column temperature on the separation of DD- and DL-diastereomers obtained from the reaction of amino acids and o-phthaldialdehyde and *N*-isobutyryl-L-cysteine derivatization reagents. Acquity BEH, CSH, and Kinetex XB stationary phases were tested with particle sizes ranging from 1.7 to 2.8 μm. The finalized method used BEH C18 column (2.1 × 50 mm, 1.7 μm) with mobile phase consisting of 0.1% H_3_PO_4_ aqueous solution (**A**) and 0.1% H_3_PO_4_ in acetonitrile (**B**). In case of [^11^C]MET, the gradient elution was accomplished by increasing the acetonitrile content from 0 to 5% with 20 min of gradient rate. For [^18^F]FET, the final acetonitrile ratio reached 40% over 25 min. At a flow rate of 0.6 ml/min the radiolabeled amino acids were separated within 10–20 min with resolution > 1.5. The methods were tested in accordance with EANM guideline on the validation of analytical methods for radiopharmaceuticals. For linearity, r^2^ > 0.997 was obtained in the concentration range of 8-180 MBq/ml, the repeatability of %Area was < 5% (RSD_%_), recovery ranged from 101.8 to 105.4%, and the limit of quantitation (LOQ) was between 9 and 13 MBq/ml.

**Conclusion:**

The novel UPLC methods meet the Ph. Eur. monograph specifications and validation requirements. The pre-column derivatization reversed-phase (RP) chromatographic protocols are suitable for determining the enantiomeric purity of [^11^C]MET and [^18^F]FET radiopharmaceuticals and can be integrated into the quality control system.

**Supplementary Information:**

The online version contains supplementary material available at 10.1186/s41181-025-00421-z.

## Background

Positron emission tomography (PET) is a widely used, minimally invasive functional imaging technique in oncology. [^11^C]methyl-L-methionine ([^11^C]MET) and *O*-(2-[^18^F]fluoroethyl)-L-tyrosine ([^18^F]FET) are important amino acid radiopharmaceuticals for the diagnosis of gliomas and brain metastases (Galldiks et al. 2023). Radiopharmaceuticals are produced as sterile solutions for intravenous administration. The batches of radiopharmaceuticals may be released for human use if they conform to specifications. Enantiomeric purity is one of the critical quality parameters of these radiopharmaceuticals. According to the monographs of the European Pharmacopeia (Ph. Eur.), both [^11^C]MET and [^18^F]FET are recommended to be tested using thin-layer chromatography (TLC) methods (Ph. Eur. monograph no. 1617 2008; Ph. Eur. monograph no. 2466 2022). The D- and L-isomers are separated on the same octadecylsilyl silica gel plate for chiral separations. While methanol-water mixture (50:50 V/V) is used for mobile phase in case of [^11^C]methionine, methanol-methylene chloride eluent (10:90 V/V) is applied for analysis of [^18^F]FET. In both cases, detection can be performed by spraying 2 g/L solution of ninhydrin in ethanol onto the TLC plates and heating them at 60 °C for 10 min. The ratio of L-isomer in the final product should be higher than 95%. However, TLC methods provide lower resolution (R_S_) and longer analysis time in relation to high-performance liquid chromatography (HPLC) (Joshi et al. 2021; Siddiq et al. 2018). It is worth noting that due to increased measurement time, [^11^C]MET could be released before performing the enantiomeric purity test to avoid significant radioactivity loss of the radiopharmaceutical.

At the same time, several liquid chromatographic methods for separating the L- and D-enantiomers of [^11^C]MET and [^18^F]FET can be found in the literature. For instance, Cheung and Ho used C8 RP Select B column (250 × 4.6 mm, 10 μm, Merck) and a mobile phase consisting of 30 mM NaOAc, 17 mM L-proline, and 8 mM Cu(OAc)_2_ for the determination of enantiomeric purity of [^11^C]methionine (Cheung and Ho 2009). The same copper-based eluent was used by Ishiwata et al. for this purpose with a reverse-phase NVC18 column (8 × 100 mm) (Ishiwata et al. 1988). Gomzina and Kuznetzova successfully applied the chiral Diacel Crownpak-CR column (0.4 × 15 cm) with an aqueous solution of HClO_4_ (pH 2.0) for the separation of D-[^11^C]MET and L-[^11^C]MET with retention times of 3.15 and 4.62 min, respectively (Gomzina and Kuznetsova  2011). Gómez et al. recommended the Astec CHIROBIOTIC T column (4.6 × 250 mm, 5 μm) and water-methanol mixture (50:50 V/V) as mobile phase (Gómez et al. 2008). Giglio et al. also proposed a chiral HPLC method for the determination of the enantiomeric purity of [^11^C]methionine. The Astec CHIROBIOTIC T column (100 × 4.6 mm 5 μm) was used with a mixture of water-methanol (20:80 V/V) at a flow rate of 1 ml/min, achieving high resolution in a run time of 7 min (Giglio et al. 2018). On the other hand, Phenomenex Chirex D penicillamine column (150 × 4.6 mm, 5 μm) was used successfully with isopropanol and 2 nM CuSO_4_ (11:89 V/V) as a mobile phase at 1 ml/min for the determination of enantiomeric purity of [^18^F]FET (Bourdier et al. 2011; Wang et al. 2019). Daicel Crownpak (+) column (3 × 150 mm) and 1.63 g/L of chloric acid-methanol (90:10 V/V) can be used for the separation of L-FET and D-FET with retention times of 11.9 and 15.8 min, respectively (Fedorova et al. 2009; Hamacher and Coenen 2002).

At the same time, α-amino acids react with *o*-phthaldialdehyde (OPA) and an optically active thiol to form diastereoisomeric derivatives, which can be separated by reverse-phase HPLC (Desai and Gal 1993). Using a fully automated instrument with a fluorescence detector, a standard octadecylsilyl stationary phase, and simple linear gradient elution conditions, the pre-column derivatization method proved to be highly reproducible, sensitive, and robust. The OPA and *N*-isobutyryl-L-cysteine (IBLC) pair is highly suitable for the simultaneous quantification of L- and D-amino acids, as well as determination of the enantiomer ratio of drugs, due to the high stability of the formed derivatives (Brückner et al. 1994). The widely used pre-column derivatization method for enantiomeric analysis of amino acids with OPA/IBLC reagents was only applied by Mueller et al. in case of a radiopharmaceutical, specifically for the separation of L-[^18^F]FET and D-[^18^F]FET (Mueller et al. 2011). The sample preparation was performed by mixing 40 µL of a solution of IBLC in 0.25 M disodium tetraborate buffer (10 mg/ml) and 20 µL of a solution of OPA in 1,4-dioxane (3.5 mg/ml) with 10 µL of [^18^F]FET. The derivatization reaction was completed in 3 min, and the sample was injected into the HPLC system. For stationary phase LiChroCART 250-4, LiChrospher 100, RP-18e, 5 μm was used. Solvent A was a mixture of acetonitrile and 0.1% trifluoroacetic acid solution (5:95 V/V), while solvent B was composed of acetonitrile and 0.1% trifluoroacetic acid solution (95:5 V/V) ratio. The following gradient elution was applied: 0–2 min 100% A, 2–20 min to 20% B, 20–40 min to 25% B, 40–60 min to 80% B. The flow rate was set to 1.2 ml/min, and the detection of non-radioactive reference compounds was performed at a wavelength of 340 nm. L-[^18^F]FET and D-[^18^F]FET derivatives were eluted at retention times of 50.7 and 51.5 min, respectively.

The aim of this work was to propose a liquid chromatographic method for the determination of the enantiomeric purity of [^11^C]MET and [^18^F]FET, as HPLC systems are commonly available in quality control laboratories and offer advantages over TLC measurements, such as automation, higher precision, and greater specificity. In addition to HPLC methods using either chiral stationary phases or mobile phases containing chiral components, commonly used reverse-phase HPLC methods have a valid rationale for implementation, as they are frequently applied for enantiomeric purity testing of amino acids via pre-column derivatization. Furthermore, the possibility of implementing a rapid chromatographic procedure using the ultra-performance liquid chromatographic technique was also investigated.

The goal of the study was to develop a liquid chromatographic method based on pre-column derivatization with OPA/IBLC system to separate the D- and L-enantiomers of [^11^C]MET and [^18^F]FET. An HPLC column produced by Core-Shell technology was used in the method development. Additionally, stationary phases with particle size of 1.7 μm were applied in an UPLC system to reduce the measurement time specific to HPLC methods. The goal of the study was to optimize the chromatographic parameters to effectively separate DD- and LD-diastereomers for the determination of enantiomeric purity of [^11^C]MET and [^18^F]FET radiopharmaceuticals.

## Methods

### Chemicals

Phosphoric acid, trifluoroacetic acid, formic acid, DL-homocysteine, DL-methionine, OPA, IBLC, sodium tetraborate, sodium hydroxide, potassium dihydrogen phosphate, and sodium acetate were obtained from Sigma. D-FET, L-FET, as well as the D- and L-forms of (2*S*)-*O*-(2′-tosyloxyethyl)-*N*-trityl-tyrosine-tert-butyl ester (TET) was purchased from ABX (Radeberg, Germany). Acetonitrile and 1,4-dioxane were supplied by VWR. All chemicals used in the experiments were analytical or HPLC grade and were used without further purification. Water was provided by a Milli-Q purification system and was controlled for organic impurity content.

### Synthesis of radiopharmaceuticals

The preparation of [^11^C]MET was started with production of C-11 isotope by GE PETtrace cyclotron according to the nuclear reaction of ^14^N(p,α)^11^C at 16.4 MeV using N_2_ gas containing 0.2% O_2_ under 10 bar pressure. The synthesis of [^11^C]methyl iodide was accomplished on a GE PETtrace MeI Microlab module via “gas phase” method. [^11^C]methionine was obtained by adding [^11^C]methyl iodide to 2 mg of DL-homocysteine and 20 mg of Al_2_O_3_, which were dispersed in 1 ml of ethanol (Schmitz et al. 1995). The purification of the raw reaction mixture was carried out by pushing 9 ml of saline through Sep-Pak C18 Plus and Sep-Pak Alumina N light cartridges (Waters). The final solution was filtered through Millex GS 0.22 μm sterile filter (Millipore). The sample contained DL-[^11^C]MET with a radioactivity concentration of up to 800 MBq/ml.

The production of [^18^F]FET was performed on Scintomics GRP 3 V synthesis module using ready-to-use GMP-compliant kits obtained from ABX (Radeberg, Germany). The synthesis proceeded via a nucleophilic substitution reaction between [^18^F]fluoride ions and D-TET or L-TET precursors (Bourdier et al. 2011). After harvesting [^18^F]fluoride ions from target water using Sep-Pak Light QMA Cartridge, the subsequent elution of the radioactivity was accomplished with.

0.075 mol/L tetrabutylammonium-bicarbonate solution. The mixture was azeotropically dried, and 10 mg of the (D)-TET precursor ((2*S*)-O-(2’-tosyloxyethyl)-*N*-trityl-D-tyrosine-*tert*-butyl ester) or 10 mg of the (L)-TET precursor ((2*S*)-O-(2’-tosyloxyethyl)-*N*-trityl-L-tyrosine-*tert*-butyl ester) was added in 2 ml acetonitrile to the reactor. The substitution reaction was carried out at 110 °C for 5 min. The synthesis of [^18^F]FET proceeded with the evaporation of acetonitrile and subsequent removal of protective groups by hydrolysis with HCl (0.2 M, 3 ml) in 8 min at 120 °C. Sep-Pak Light Alumina N, Sep-Pak WAX, and Sep-Pak HLB Cartridges were used for purification of the raw reaction mixture. The product was eluted from the cartridges with 12 ml of ethanol-water mixture (1:19 V/V). The final solution was filtered through 0.22 μm Millex-GS Syringe-Driven Filter Unit (Millipore). The synthesis time was 55 min, and the radioactivity of the obtained product was up to 2 GBq.

### Chromatography

Waters Acquity *I* Class UPLC system was used to perform chromatographic measurements. UV-Vis detector was employed for detection of non-radioactive reference materials, while radioactive components were identified using an in-house constructed radioactive detector based on a photomultiplier tube (PMT) coupled to a plastic scintillator (Hamamatsu). Empower 3 software was used for acquisition and evaluation of the measurements. The method development involved the following stationary phases: Acquity BEH C18 column (2.1 × 100 mm, 1.7 μm, Waters); Acquity BEH C18 column (2.1 × 50 mm, 1.7 μm Waters), Acquity CSH C18 column (2.1 × 100 mm, 1.7 μm, Waters), and Kinetex XB-C18 column (4.6 × 50 mm, 2.6 μm, Phenomenex). A column manager was used to adjust the column temperature. The injection volume ranged from 1 to 5 µl. The analysis of non-radioactive samples was repeated three times, and the average chromatographic values were used for evaluating the results. The RSD values of the Area% for L-[^11^C]MET and L-[^18^F]FET were calculated based on a repeatability study of the finalized methods involving analyses performed by different operators.

### Sample preparation

Solutions of non-radioactive reference materials, namely DL-methionine and DL-FET were prepared at a concentration of 1 mg/ml. Radioactive tracers were used at concentrations of 50–1000 MBq/ml. Samples for chromatographic measurements were prepared by mixing 10 µL of amino acid solution, 60 µL of solution of IBLC in 0.25 M disodium tetraborate buffer (10 mg/ml), and 30 µL of OPA solution in 1,4-dioxane (3.5 mg/ml). The resulting mixtures were analyzed after 3 min of storage to complete the derivatization reaction.

### Method development plan for chromatographic analysis

In this work, a well-known chromatographic method for the separation of amino acid enantiomers, based on pre-column derivatization using the IBLC/OPA system was applied for the determination of enantiomeric purity of [^11^C]MET and [^18^F]FET. Both D- and L-forms of the radiopharmaceuticals at nanomolar concentrations react with the derivatization reagents to form diastereomers, which can be separated by reverse-phase liquid chromatography. Method development was conducted using non-radioactive DL-methionine and DL-FET for reference materials to optimize chromatographic conditions. However, the final procedures were validated using radioactive samples of amino acids labeled with F-18 and C-11 isotopes. The separations were performed on stationary phases suitable for UPLC conditions, including Acquity BEH C18 (2.1 × 100 mm, 1.7 μm, BEH100); Acquity BEH C18 (2.1 × 50 mm, 1.7 μm, BEH50) and Acquity CSH C18 (2.1 × 100 mm, 1.7 μm, CSH). Additionally, Kinetex XB-C18 column (4.6 × 50 mm, 2.6 μm) was used to test the efficiency of the stationary phase produced by Core-Shell technology. The optimization protocol focused on three main mobile phases, namely the following binary mixtures were used: 0.1% H_3_PO_4_ in water/0.1% H_3_PO_4_ in acetonitrile, 0.1% TFA in water/0.1% TFA in acetonitrile, and 0.1% HCOOH in water/0.1% HCOOH in acetonitrile. The low pH of the eluents was chosen to keep the diastereomers and silanol groups of stationary phases in ion suppressed form to minimize the number of interactions between the components and surface of the silica gel, thus achieving high kinetic efficiency. Additionally, the separation of the enantiomers was also studied at higher pH conditions. Furthermore, the flow rate and column temperature were optimized to achieve peak resolution higher than 1.5. The measurement of non-radioactive samples was repeated three times, and the average resolution values were used for the evaluation of the results. It was observed that the standard deviation (SD) did not exceed 0.03.

## Results

### [^11^C]MET

The development of a liquid chromatographic method for determination of enantiomeric purity of [^11^C]methionine was started with study of the effect of gradient time on the resolution of DD- and DL-diastereomers. Initially, the stationary phase was eluted with an aqueous mobile phase and after sample injection the acetonitrile content in the eluent was increased from 0 to 95%. The gradient time varied between 2 and 25 min (Table 1).

**Table 1:** Elution profile of the optical isomers of methionine

Time (min)	0.1% H_3_PO_4_ in H_2_O (per cent V/V)	0.1% H_3_PO_4_ in ACN (per cent V/V)
0	100	0
0 (2, 5, 10, 15, 20, 25)	100 → 5	0 → 95

Time (min)	0.1% TFA in H_2_O (per cent V/V)	0.1% TFA in ACN (per cent V/V)
0	100	0
0 (2, 5, 10, 15, 20, 25)	100 → 5	0 → 95

Time (min)	0.1% HCOOH in H_2_O (per cent V/V)	0.1% HCOOH in ACN (per cent V/V)
0	100	0
0 (2, 5, 10, 15, 20, 25)	100 → 5	0 → 95

**Fig. 1:**
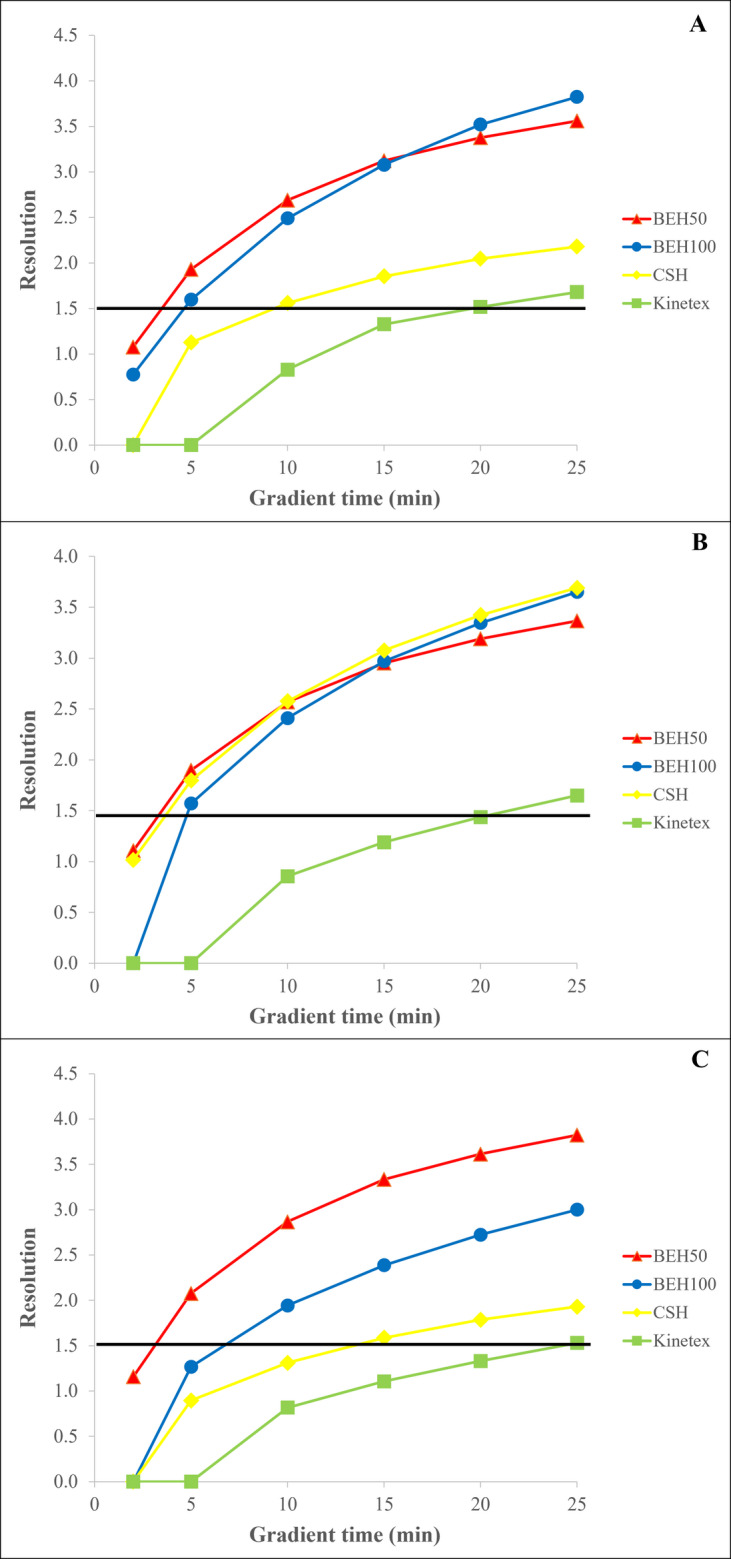
Effect of gradient time on the separation of D- and L-methionine using phosphoric acid (**A**), trifluoroacetic acid (**B**), and formic acid (**C**) for the preparation of mobile phases

**Fig. 2:**
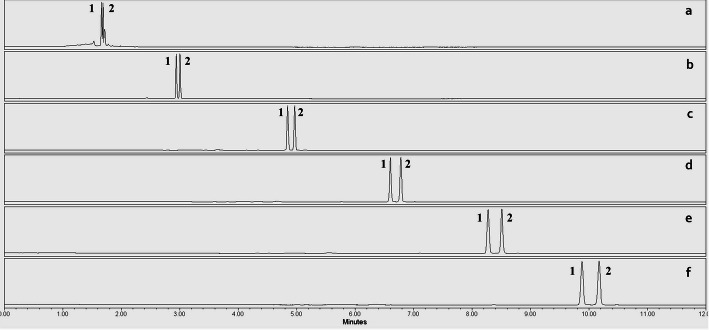
Separation of DD- and DL-diastereomers of methionine derivatives on the BEH 50 column at 2 (**a**), 5 (**b**), 10 (**c**), 15 (**d**), 20 (**e**), and 25 (**f**) minutes of gradient time using phosphoric acid-based eluents. (**1**: L-MET; **2**: D-MET)

It was observed that Kinetex column could hardly provide baseline resolution, with R_S_>1.5 being achieved only at 25 min of gradient time for all examined acidic eluents (Fig. 1). Among the mobile phases tested, the formic acid-based eluent yielded the poorest results, while phosphoric acid and TFA solutions achieved a peak resolution of up to 1.65. Thus, within the studied gradient time range, the core-shell stationary phase failed to effectively separate the diastereomers. Among columns with a particle size of 1.7 μm, the CSH stationary phase provided moderate resolution compared to the BEH columns when using phosphoric and formic acid-based eluents. Baseline separation of the peaks was achieved at 10 and 15 min of gradient time, respectively. The peak resolution increased to 2.2 with the last eluted peak having a retention time of 12.9 min. In contrast, when TFA was used for the preparation of the acidic eluents, the resolution was comparable to that of the BEH columns, reaching 3.7. This improved separation is likely due to the controlled positive charge of the stationary phase and the influence of TFA anions. However, the BEH columns ultimately provided the best results in separation of diastereomers. Acceptable peak resolution was achieved over a wide range of gradient times with a maximum of 3.8 (Fig. 2). Baseline resolution was achieved from 5 min of gradient time, significantly reducing the retention time of the last eluting component to less than 3 min. As a result, the overall measurement time was shortened, enabling fast separation of the enantiomers using the UPLC technique. While similar resolution could be obtained with both phosphoric acid and TFA-based eluents, formic acid solutions caused a twofold reduction in peak resolution. Based on these findings, the BEH50 column and the gradient time of 5 min were selected for further optimization of the diastereomer separation.

The role of mobile phase pH on the separation of diastereomers was also investigated. In addition to the previously used phosphoric acid-based eluents, two other eluent pairs were tested: 10 mM sodium acetate solution (pH 5) and 10 mM sodium acetate (pH 5)-acetonitrile mixture (1:4 V/V), as well as 10 mM sodium phosphate solution (pH 7) and 10 mM sodium phosphate (pH 7)-acetonitrile mixture (1:4 V/V). The BEH50 column was conditioned with aqueous sodium acetate or sodium phosphate solutions, and after the sample injection the buffer-acetonitrile binary mixtures were added with gradient time of 4 min. The acetonitrile ratio was kept the same as in the phosphoric acid-based eluents. Our results showed that the resolution of 2.07 achieved at pH 2 decreased to 1.95 at pH 5 and 1.45 at pH 7 (Table 2). Therefore, a highly acidic pH is necessary for the mobile phase to keep the silanol groups of the stationary phase and the diastereomers in ion-suppressed forms, reducing interactions and improving kinetic efficiency and resolution. For subsequent measurements the pH of mobile phases was maintained at 2.

**Table 2:** Effect of pH of the eluents, flow rate, and column temperature on peak resolution of DL-enantiomers of methionine using BEH50 column

Parameter			Resolution
pH	Flow, (ml/min)	Temp., (°C)	
2	0.6	25	2.07 ± 0.01
5	0.6	25	1.95 ± 0.01
7	0.6	25	1.45 ± 0.01
2	0.4	25	1.97 ± 0.01
2	0.6	25	2.07 ± 0.01
2	0.8	25	2.25 ± 0.01
2	0.6	25	2.07 ± 0.01
2	0.6	30	2.28 ± 0.01
2	0.6	40	2.34 ± 0.01

Method development proceeded with an investigation of the effects of flow rate of the eluent and column temperature on peak resolution. It was observed that increasing the flow rate resulted in higher peak resolution. At a flow rate of 0.4 ml/min, the resolution was 1.97, while increasing the flow rate to 0.8 ml/min raised the resolution to 2.25 (Table 2). However, due to significant system pressure flow rate of 0.6 ml/min was selected for subsequent measurements, providing a peak resolution of 2.07. Finally, the column temperature was varied from ambient temperature to 30 °C and 40 °Cto examine the impact on separation. It was found that higher peak resolutions were achieved at higher temperatures with resolutions of 2.28 and 2.34 at 30 °C and 40 °C, respectively (Table 2).

Based on the results of the method development, the following conditions were selected for the finalized chromatographic method to separate D- and L-methionine. Acquity BEH C18 column (2.1 × 50 mm, 1.7 μm, Waters) was selected for the stationary phase. Eluent A was 0.1% H_3_PO_4_ in water, and eluent B was 0.1% H_3_PO_4_ in acetonitrile. The gradient elution profile was as follows: 0 min‒100% A, 5 min‒5% A. The flow rate was set to 0.6 ml/min, and the column temperature was maintained at ambient. The injected volume was 1 µl, and the detection wavelength was 333 nm. The measurement could be completed in 4 min with a peak resolution of 2.07.

**Fig. 3:**
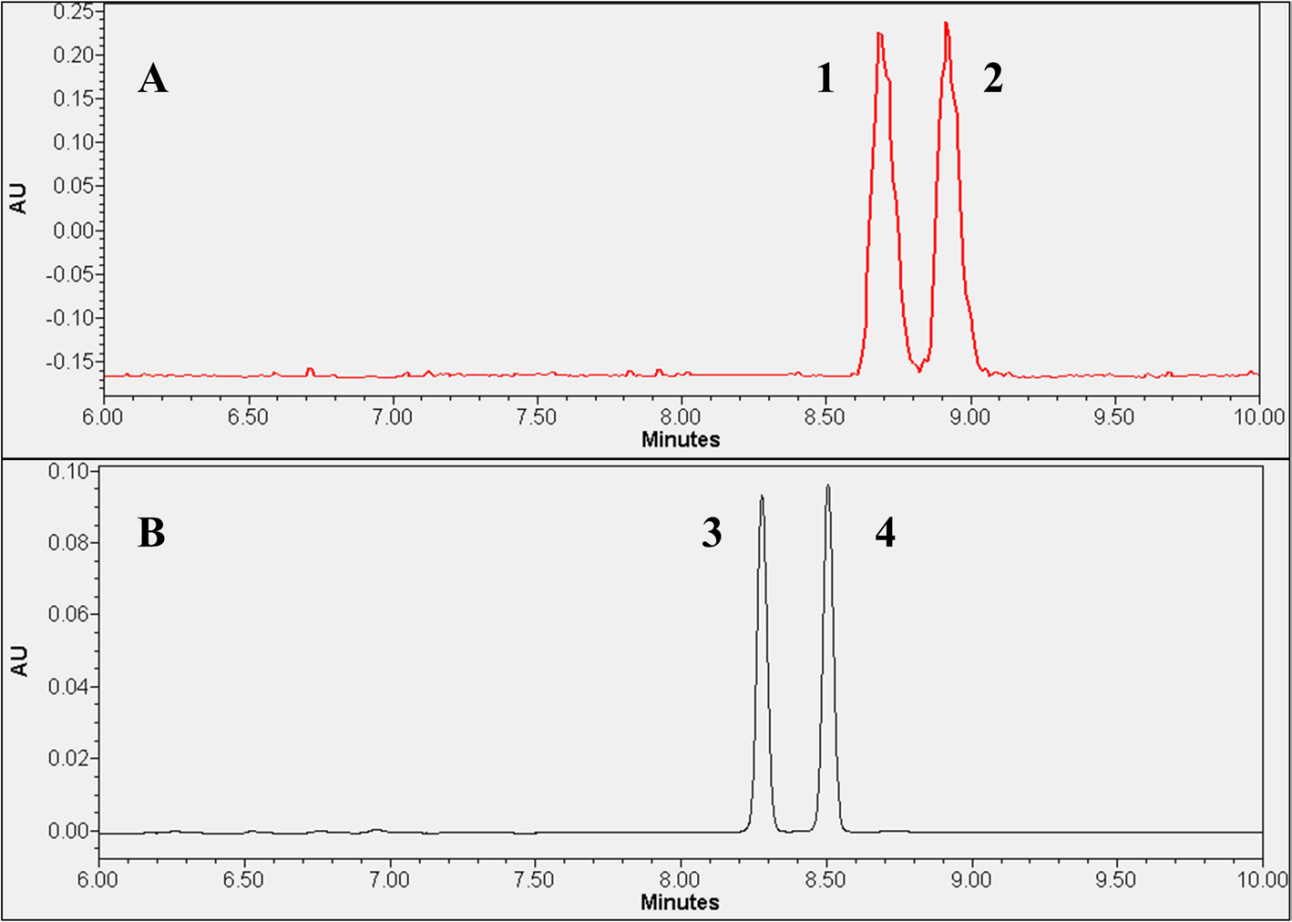
Chromatograms obtained using finalized method for the separation of D- and L-enantiomers of methionine. (**A**: Radioactive chromatogram; **B**: UV chromatogram; **1**: L-[^11^C]MET; **2**: D-[^11^C]MET; **3**: L-[^12^C]MET; **4**: D-[^12^C]MET)

In the final step, the developed UPLC method was adapted for measurement of radioactive enantiomers of methionine. Considering that the dimensions of the in-house constructed radioactivity flow-through detector and the UV-Vis detector show significant differences, peak broadening was expected for D- and L-[^11^C]MET. Indeed, the radiolabeled diastereomers were separated with an acceptable resolution of 1.5 only at gradient time of 20 min (Fig. 3A), whereas the non-radioactive pairs achieved a resolution of 3.38 (Fig. 3B). As a result, the measurement time increased to 10 min. The suitability of the novel method for separation of D- and L-[^11^C]MET was validated according to the EANM guidelines on the validation of analytical methods for radiopharmaceuticals (Gillings et al. 2020). Validation results showed that for linearity, the regression coefficient (r^2^) ranged from 0.9972 to 0.9989. The measurement range was 9-180 MBq/ml, with a relative standard deviation (RSD_%_) of %Area of 4.4%. The recovery of radioactive components was 105.4%, and the limit of quantification (LOQ) was 9 MBq/ml (Supplementary information, Fig. S1–S4).

## [^18^F]FET

The development of a chromatographic method for determination of the enantiomeric purity of [^18^F]FET was started with optimization of the mobile phase. This was achieved by studying the effect of gradient time on the resolution of DD- and DL-diastereomers of non-radiolabeled tyrosine using aqueous and acetonitrile solutions of phosphoric acid, TFA, and formic acid. The initial eluent composition was maintained at 100% aqueous acidic solution, and after sample injection, the acetonitrile content was increased from 0 to 40%. The gradient time ranged from 2 to 25 min (Table 3).

**Table 3:** Elution profile of the optical isomers of FET

Time (min)	0.1% H_3_PO_4_ in H_2_O (per cent V/V)	0.1% H_3_PO_4_ in ACN (per cent V/V)
0	100	0
0 (2, 5, 10, 15, 20, 25)	100 → 60	0 → 40

Time (min)	0.1% TFA in H_2_O (per cent V/V)	0.1% TFA in ACN (per cent V/V)
0	100	0
0 - (2, 5, 10, 15, 20, 25)	100 → 60	0 → 40

Time (min)	0.1% HCOOH in H_2_O (per cent V/V)	0.1% HCOOH in ACN (per cent V/V)
0	100	0
0 (2, 5, 10, 15, 20, 25)	100 → 60	0 → 40

**Fig. 4:**
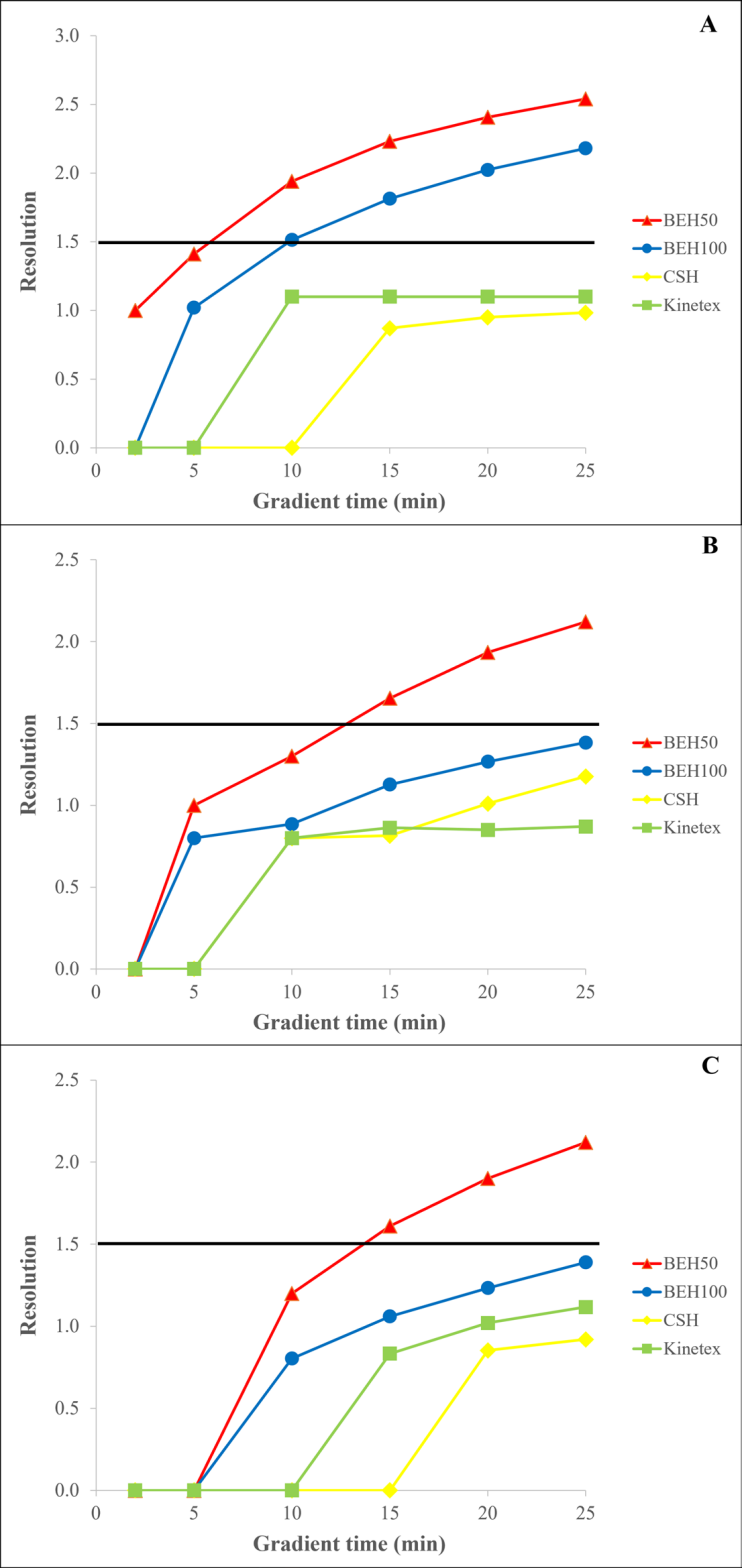
Effect of gradient time on the separation of D- and L-FET using phosphoric acid (**A**), trifluoroacetic acid (**B**), and formic acid (**C**) for the preparation of mobile phases

According to our results, the Kinetex column could not provide acceptable peak resolution, as the minimum criteria of 1.5 were not achieved even at 25 min of gradient time regardless of the acidic solution used (Fig. 4). Interestingly, baseline resolution was also not achieved with the CSH column, and it showed lower resolution than the Kinetex column when using formic and phosphoric acid. The BEH100 stationary phase could not meet the resolution criteria with formic acid and TFA. Acceptable baseline resolution was only obtained with phosphoric acid solutions from 10 min of gradient time. The highest resolution of 2.2 could be achieved at a gradient time of 25 min, and the last eluting component had a retention time of 12.5 min. In contrast, the BEH50 column provided baseline resolution only after 15 min of gradient time when using TFA and formic acid-based eluents, with a maximum resolution of 2.1. However, R_S_=2 was achieved at 10 min of gradient time with phosphoric acid solutions, and the resolution could be increased to 2.5 by increasing the acetonitrile content in the mobile phase (Supplementary information, Fig. S5). Therefore, the BEH50 column was selected for further optimization measurements, as it provided the best separation results for the enantiomers of FET, achieving baseline resolution across a wide range of gradient times, while reducing the chromatographic run time to 8 min.

The effect of pH of the mobile phase on the separation of diastereomers was also examined. A 10 mM sodium acetate (pH 5) solution, 10 mM sodium acetate (pH 5)-acetonitrile mixture (1:4 V/V), and 10 mM sodium phosphate solution (pH 7) as well as 10 mM sodium phosphate (pH 7)-acetonitrile mixture (1:4 V/V) were used. The stationary phase was conditioned with aqueous solutions, and after sample injection the buffer-acetonitrile mixtures were added with gradient rate of 8 min. The acetonitrile ratio throughout the elution profile was consistent with that used in the experiments with phosphoric acid-based solutions. Our results indicated that the resolution of 1.94 obtained at pH 2 decreased to 1.45 at pH 5 and 0.84 at pH 7 (Table 4). These findings suggest that maintaining a highly acidic pH of the is essential to suppress the ionization of silanol groups on the stationary phase to prevent interaction with the diastereomers, thereby improving kinetic efficiency and resolution. Therefore, for subsequent measurements, the mobile phase pH was maintained at 2.

**Table 4:** Effect of pH of the eluents, flow rate, and column temperature on the peak resolution of FET enantiomers using BEH50 column

Parameter			Resolution
pH	Flow, (ml/min)	Temp., (°C)	
2	0.6	25	1.94 ± 0.01
5	0.6	25	1.45 ± 0.01
7	0.6	25	0.85 ± 0.01
2	0.4	25	1.77 ± 0.03
2	0.6	25	1.94 ± 0.01
2	0.8	25	2.11 ± 0.03
2	0.6	25	1.94 ± 0.01
2	0.6	30	2.11 ± 0.01
2	0.6	40	2.20 ± 0.01

The method development continued with a study of the effect of flow rate of the eluent and column temperature on peak resolution. It was observed that increasing the flow rate resulted in higher peak resolution. At 0.4 ml/min the resolution was 1.77, while at 0.8 ml/min the R_S_ increased to 2.11 (Table 4). Considering the increased system pressure, a flow rate of 0.6 ml/min was chosen for subsequent measurements, providing an acceptable peak resolution of 1.94. Finally, the column temperature was increased from ambient to 30 °C and 40°C to improve the separation of the components. The resulting peak resolutions were 2.11 and 2.20, respectively (Table 4). Consequently, higher temperatures allowed for more effective separation.

Based on the results from method development, the following conditions were chosen for the finalized chromatographic method. Acquity BEH C18 column (2.1 × 50 mm, 1.7 μm, Waters) was used for the stationary phase. Eluent A was 0.1% H_3_PO_4_ in water, and eluent B was 0.1% H_3_PO_4_ in acetonitrile. The gradient elution profile was as follows: 0 min‒100% A, 10 min‒60% A. The flow rate was adjusted to 0.6 ml/min, and column temperature was ambient. The injected volume was 1 µl, and the detection wavelength was 333 nm. The measurement could be completed in 10 min with a peak resolution of 2.19.

**Fig. 5:**
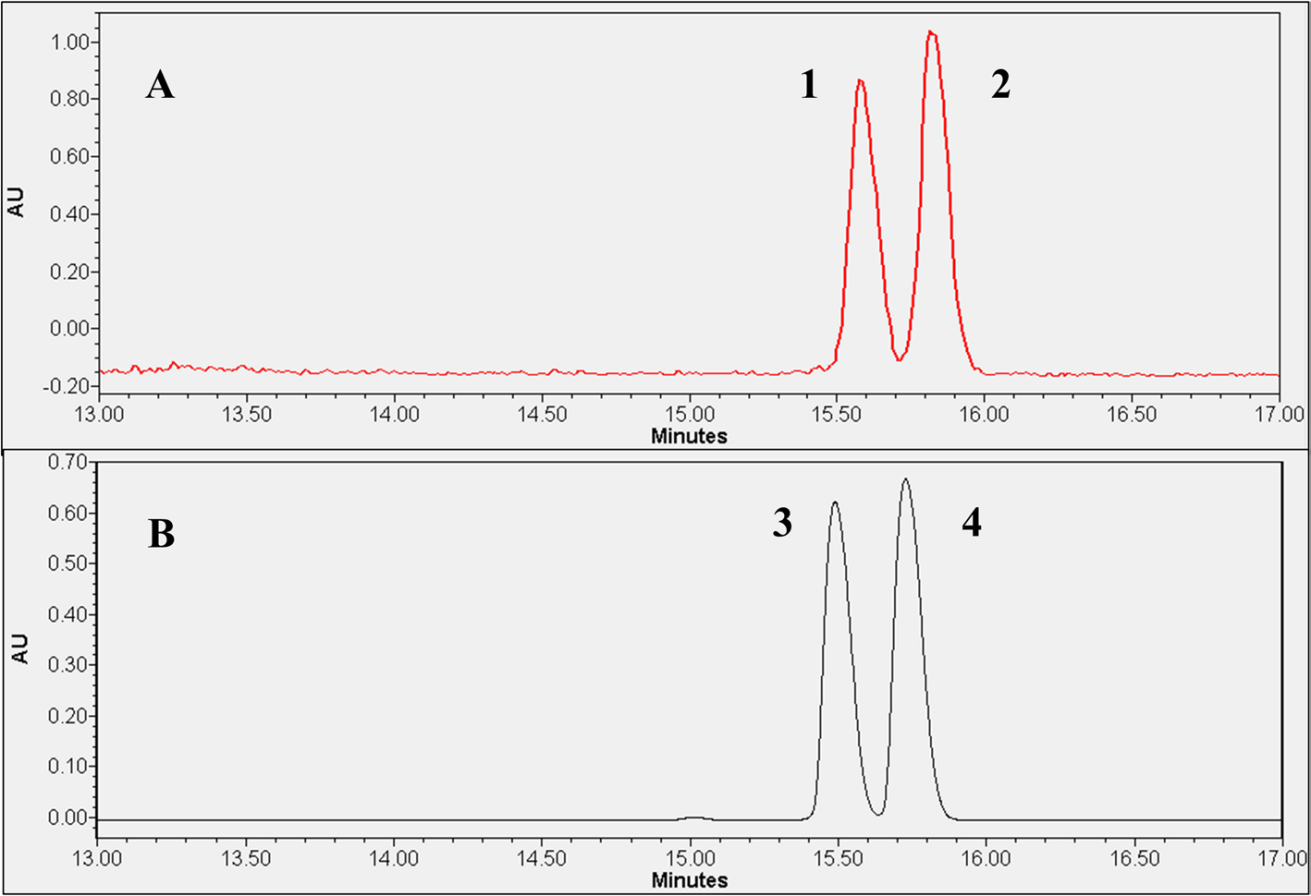
Chromatograms obtained by the finalized method for the separation of D- and L-enantiomers of FET. (**A**: radioactive chromatogram; **B**: UV chromatogram; **1**: L-[^18^F]FET; **2**: D-[^18^F]FET; **3**: L-[^19^F]FET; **4**: D-[^19^F]FET)

To apply the parameters of the developed UPLC-UV method for the measurement of optical isomers of [^18^F]FET, a compromise had to be made due to the peak broadening of D- and L- enantiomers in the radioactive flow-through detector. A gradient rate of 25 min was required to separate the radiolabeled diastereomers with resolution of 1.7 (Fig. 5). Consequently, the measurement time also increased to 17 min. The validation of the radiochromatographic method was performed according to the protocol used for [^18^F]FET. Based on the results obtained, the linearity (r^2^) ranged from 0.9978 to 0.9997, the RSD% of %Area was 1.1%, recovery was 101.8%, and the LOQ was 13 MBq/ml (Supplementary information, Figs. S6–S9).

## Discussion

Several quality control parameters should be evaluated to assess the quality of radioactive preparations. The pre-release of the radiopharmaceuticals can be performed based on critical analytical results (Hendrikse et al. 2022). Due to the rapid radioactive decay of short-lived nuclides, some tests can be performed after the release of the preparations to avoid considerable loss of radioactivity in the batches. To release [^11^C]MET and [^18^F]FET radiopharmaceuticals for human use, quality control measurements should be performed within a short timeframe.

Enantiomeric purity is a required criterion for the release of [^11^C]MET and [^18^F]FET radiopharmaceuticals. The European Pharmacopeia recommends the use of thin-layer chromatography to determine enantiomeric purity in both cases (Ph. Eur. monograph no. 1617 2008; Ph. Eur. monograph no. 2466 2022). The TLC test has several advantages, namely that it provides simple and rapid analysis; thin-layer scanners equipped with radio-detectors are non-complex instruments and are accessible to most radiopharmaceutical production sites manufacturing^18^F-tracers. Consequently, the radio-TLC method seems to offer a convenient way to perform the enantiomeric purity test. On the other hand, thin-layer chromatography presents certain challenges, such as moderate resolution and limited quantitative accuracy compared to liquid chromatography. Indeed, special care should be taken during the preparation and storage of mobile phase, developing reagents, and reference solutions to achieve acceptable peak resolution and reliable results. The TLC octadecylsilyl silica gel plate for chiral separations should also be handled with care to ensure acceptable results.

The accuracy and reliability of the enantiomeric purity test can be enhanced by using liquid chromatography. The analysis can be performed with excellent resolution compared to the TLC method, and automation is available to achieve high reproducibility. The cost of the HPLC system is not much higher than that of TLC scanners and it is commonly used in radiochemical laboratories. In this context, the use of liquid chromatography is more appropriate for production sites with trained personnel. The direct use of chiral stationary phases presents several drawbacks, namely that the columns are expensive and sensitive to metal contamination. Therefore, the widely used pre-column derivatization RP-LC method could be a prospective approach for the indirect determination of enantiomeric purity of [^11^C]MET and [^18^F]FET.

In this study, the OPA/IBLC derivatization pair was used to produce D, D- and D, L-diastereomers of the optically active amino acids, which could be separated on octadecylsilyl (ODS) columns using robust reverse-phase chromatography, thereby avoiding the challenges associated with chiral stationary phases. Derivatization reagents can be prepared easily and used for several weeks, provided they are stored at -20 °C. At higher temperatures, OPA and IBLC solutions degrade, and derivatization reaction does not occur. The sample preparation is less complex compared to that given in the Ph. Eur. methods. After mixing the test solution with OPA/IBLC reference solutions, the mixture can be injected into the analytical column immediately. Additionally, the system suitability test with non-radioactive components can be performed more easily than with the TLC method. The procedure can be automated, and the evaluation of the chromatograms is also simple. Due to the optimized mobile phases and gradient profile, the separation of optical isomers of [^11^C]MET and [^18^F]FET can be achieved with good resolution. This may be problematic in some instances during TLC procedures due to broader peaks and moderate resolution. Obviously, the developed pre-column derivatization method is highly accurate and precise. In this paper, analytical columns suitable for UPLC techniques were evaluated for the separation of diastereomers. Stationary phases with a particle size of 1.7 μm were used to achieve accurate separation and reduced analysis time, typical of HPLC. Columns produced using the BEH technology were found to be more effective than those produced with the CSH version. Interestingly, the shorter BEH50 column provides higher resolution than the BEH100 column, which is double the length; additionally, the analysis time can be reduced almost by half. Surprisingly, the Kinetex column prepared using Core-Shell technology did not meet the requirements and provided only moderate peak resolution. Additionally, the composition of the mobile phase determines the peak resolution. In this context, the eluent prepared with phosphoric acid proved to be more effective than those prepared with TFA or formic acid. A pH of 2 proved to be optimal for separation, and a flow rate of 0.6 ml/min is recommended for elution. The finalized UPLC methods for the enantiomeric purity testing of [^11^C]MET and [^18^F]FET provide a convenient and reliable analytical procedure. A typical reverse-phase liquid chromatographic elution can be performed without requiring sophisticated procedures. The only difference from conventional LC procedures is the derivatization step in sample preparation, which can be accomplished easily. The main advantage of the developed methods is the reduced analysis time compared to TLC methods. For [^11^C]MET, the UPLC analysis can be completed in 10 min, whereas thin-layer chromatography requires over 20 min. This indicates that using LC methods, enantiomeric purity results can be obtained prior to the release of [^11^C]MET. In this manner, the efficiency and safety of the radiopharmaceutical can be enhanced, which is the most important consideration for preparations intended for human use.

It is also worth discussing the relationship between the performance of the proposed UPLC methods and the procedures based on chiral stationary phases. The retention times of L-[^11^C]MET and D-[^11^C]MET are approximately 3 and 5 min, respectively, according to the literature (Gomzina and Kuznetsova  2011; Gómez et al. 2008; Giglio et al. 2018). The peak resolution exceeds 2, and the measurement time is under 7 min when using the CHIROBIOTIC T column. The proposed UPLC method provides the separation of the enantiomers with a resolution of 1.5 and retention times ranging from 8.5 to 9.0 min. Considering the time required for the derivatization procedure, the measurement can be completed in 12 min. In case of [^18^F]FET, the measurement can be performed in 25 min with good peak resolution using a Chirex D column (Bourdier et al. 2011; Wang et al. 2019). At the same time, the presented derivatization method provides a resolution of 1.7 for L-[^18^F]FET and D-[^18^F]FET within a 20 min measurement time. Consequently, although the resolution of the enantiomer peaks is lower than that obtained with chiral stationary phases, the measurement times are comparable to those reported in the literature.

The recommended liquid chromatographic methods are suitable for use with UPLC systems. Obviously, this technique is not as widely used as HPLC due to its higher initial and maintenance costs. However, the procedure can be easily transferred to HPLC systems. Additionally, the analysis time can approach that of UPLC when Core-Shell columns with a particle size of 2.6–2.7 μm are used, as they provide higher efficiency compared to conventional HPLC columns. To promote the wider adoption of the pre-column derivatization method, further investigations should be conducted in this area.

## Conclusions

In this work, we successfully developed two chromatographic methods for the separation of L- and D-isomers of [^11^C]MET and [^18^F]FET, based on pre-column derivatization using the IBLC/OPA system for determination the enantiomeric purity of these radiopharmaceuticals. The results of the method development revealed that the Kinetex XB-C18 (4.6 × 50 mm, 2.6 μm) column produced by Core-Shell technology, was not suitable for the separation of the optical isomers. On the other hand, the Acquity CSH C18 (2.1 × 100 mm, 1.7 μm) column was only effective for in methionine analysis. Unambiguously, the Acquity BEH C18 column (2.1 × 50 mm, 1.7 μm) was found to be the best choice for achieving the highest resolution across a wide range of gradient times using mobile phases based on TFA, phosphoric, and formic acid. Therefore, for the finalized methods, the Acquity BEH C18 column (2.1 × 50 mm, 1.7 μm) is recommended for the effective separation of the obtained D, D- and D, L-diastereomers of amino acids using eluents composed of 0.1% H_3_PO_4_ in water (A) and 0.1% H_3_PO_4_ in acetonitrile (B). The gradient elution profile for [^11^C]MET was as follows: 0 min 100% A, 20 min 5% A. In contrast, for [^18^F]FET, the mobile phase gradient was modified to: 0 min 100% A, 25 min 40% A. The flow rate was set to 0.6 ml/min, and the column was maintained at an ambient temperature. The enantiomers of the radiolabeled amino acids were separated at baseline, and the analysis time ranging from 10 to 20 min. Validation of the novel methods provided the following results: linearity, r^2^ > 0.997; repeatability of %Area, RSD_%_<5%; recovery = 101.8-105.4% and LOQ = 9–13 MBq/ml.

## Supplementary Information

Below is the link to the electronic supplementary material.

Supplementary Material 1

## Data Availability

The datasets used and/or analyzed during the current study are available from the corresponding author on reasonable request.

## Abbreviations

[^11^C]MET: [^11^C]methyl-L-methionine
[^18^F]FET: *O*-(2-[^18^F]fluoroethyl)-L-tyrosine
ACN: Acetonitrile
BEH: Ethylene bridged hybrid
CSH: Charged surface hybrid
EANM: European Association of Nuclear Medicine
HPLC: High-performance liquid chromatography
IBLC: *N*-isobutyryl-L-cysteine
LOQ: Limit of quantification
ODS: Octadecylsilyl
OPA: *O*-phthaldialdehyde
PET: Positron emission tomography
Ph. Eur.: European Pharmacopeia
PMT: Photomultiplier tube
r^2^: Regression coefficient
R_S_: Resolution
RSD_%_: Relative standard deviation
SD: Standard deviation
TET: (2*S*)-*O*-(2′-tosyloxyethyl)-*N*-trityl-tyrosine-tert-butyl ester
TFA: Trifluoroacetic acid
TLC: Thin-layer chromatography
UPLC: Ultra-performance liquid chromatographic
UV-Vis: Ultraviolet-visible detector
