# Supplementary material for: Liquid chromatographic determination of enantiomeric purity of [11C]methyl-L-methionine and O-(2-[18F]fluoroethyl)-L-tyrosine by pre-column derivatization with o-phthaldialdehyde and N-isobutyryl-L-cysteine
Source: EJNMMI Radiopharm Chem. 2026 Jan 7;11:9. doi: 10.1186/s41181-025-00421-z (PMC12873027; doi:10.1186/s41181-025-00421-z)
Supplement: Supplementary file 1 — Supplementary Material 1 [file 41181_2025_421_MOESM1_ESM.docx]

**Supplementary information**

**Liquid chromatographic determination of enantiomeric purity of [^11^C]methyl-L-methionine and
*O*-(2-[^18^F]fluoroethyl)-L-tyrosine by pre-column derivatization with *o*-phthaldialdehyde and
*N*-isobutyryl-L-cysteine**

Viktória Forgács, Viktória Balla, Viktória Csonka, Dezső Szikra, Dániel Szücs, Enikő Németh, Zita Képes, György Trencsényi, István Jószai*

*Division of Nuclear Medicine and Translational Imaging, Department of Medical Imaging, Faculty of Medicine, University of Debrecen, 98 Nagyerdei St., H-4032 Debrecen, Hungary*

* Corresponding author. Tel.: + 36 52 255510. fax: +36 52 255000

*E-mail address:*joszai.istvan@med.unideb.hu (I. Jószai).

**Fig. S1** Linearity test of L-[^11^C]MET (Area)

**Fig. S2** Linearity test of D-[^11^C]MET (Area)

**Fig. S3** Linearity test of L-[^11^C]MET (Height)

**Fig. S4** Linearity test of D-[^11^C]MET (Height)

**Fig. S5** Separation of DD- and DL-diastereomers of FET derivatives on BEH 50 column at various gradient time using phosphoric acid-based eluents. (a: 25 min; b: 20 min;
c: 15 min; d: 10 min; e: 5 min; f: 2 min).

**Fig. S6** Linearity test of L-[^18^F]FET (Area)

**Fig. S7** Linearity test of D-[^18^F]FET (Area)

**Fig. S8** Linearity test of L-[^18^F]FET (Height)

**Fig. S9** Linearity test of D-[^18^F]FET (Height)
